# Prevalence and Incidence of Human Papillomavirus (HPV) Infection Before and After Pregnancy: Pooled Analysis of the Control Arms of Efficacy Trials of HPV-16/18 AS04-Adjuvanted Vaccine

**DOI:** 10.1093/ofid/ofz486

**Published:** 2019-12-04

**Authors:** Jing Chen, Kusuma Gopala, Akarsh Puthatta, Frank Struyf, Dominique Rosillon

**Affiliations:** 1 GSK, Singapore, Singapore; 2 GSK, Bangalore, India; 3 GSK, Wavre, Belgium

**Keywords:** human papillomavirus, incidence, pregnancy, prevalence, risk factor

## Abstract

**Objective:**

Data on human papillomavirus (HPV) prevalence around pregnancy were inconsistent. We assessed HPV prevalence before and after pregnancy, HPV incidence after pregnancy, and risk factors for HPV infection.

**Method:**

Data from 15 754 women in control arms of 5 AS04-HPV-16/18 vaccine efficacy trials were analyzed, including 3001 women with at least 1 pregnancy. Results of HPV deoxyribonucleic acid testing on cervical samples were available. We analyzed risk factors, including age, region, pregnancy and its outcomes, duration from pregnancy resolution to collection of first postresolution cervical sample, previous HPV infection, cigarette smoking, and number of sexual partners with Cox regression.

**Results:**

Prevalence of high-risk oncogenic (hr)-HPV types was similar before and after pregnancy (20.8% vs 19.8%). Incidence of hr-HPV was 40.1 per 1000 person-years (95% confidence interval [CI], 23.4–64.2) at 0–3 months, 266.7 (95% CI, 217.4–323.7) at 3–6 months, and 95.7 (95% CI, 83.9–108.7) at >6 months after pregnancy. Risk factors associated with HPV infection after pregnancy are previous HPV infection, elective abortion, and younger age at pregnancy resolution.

**Conclusions:**

Pregnancy could not be confirmed as a risk factor for HPV infection in this population despite an increased incidence detected 3–6 months after pregnancy resolution. Most women remained HPV negative after pregnancy.

**Clinical Trial Registration:**

NCT001226810 (HPV-008 trial), NCT00294047 (HPV-015 trial), NCT00316693 and NCT00929526 (HPV-032/063 trials), and NCT00779766 (HPV-039 trial).

## INTRODUCTION

Persistent human papillomavirus (HPV) infection is a prerequisite for cervical cancer [1], a disease that kills 266 000 women worldwide each year [2]. About 82% of all invasive cervical cancer cases are accounted for by persistent infections with the oncogenic or high-risk (hr) HPV types 16, 18, 31, 33, and 45 [3]. As HPV is the most common sexually transmitted infection globally, most women become infected at some point in their life, although most of these infections are transient with only a small proportion becoming persistent [4]. The risk of having HPV infection is highest in young, sexually active women and increases significantly with each additional sexual partner [5]. Several studies suggest that pregnancy is a risk factor for HPV infection due to changes in hormonal levels that alter the immune system [6–9], but findings from various studies have been inconsistent [4, 10–13]. Only a few studies, including (for the most part) small cohorts, followed pregnant women after delivery. It also was unclear whether pregnancy or other risk factors (for example, tissue damage to the cervix during delivery, sexual behavior, or age) could explain any differences in HPV detection observed between nonpregnant, pregnant, and postpartum women. If women indeed are more prone to HPV infection during or shortly after pregnancy, additional prevention measures to vaccination, such as increased cervical screening and postpartum follow up, may be needed, especially because parity is known as a risk factor for cervical cancer [8, 14].

The efficacy of vaccination against persistent HPV infection and the prevention of cervical intraepithelial neoplasia have been demonstrated widely [15, 16]. In many countries, vaccination against the most common hr-HPV types has been implemented in routine immunization schedules for preadolescent girls [17]. Extensive clinical trials showed that the HPV-16/18 AS04-adjuvanted vaccine also is effective against development of HPV-16/-18 associated cervical intraepithelial neoplasia in adult women. In addition, the vaccine induced a sustained immune response, reduced HPV incidence, and cross-protected against other hr-HPV types [15–19].

The current study, which is an analysis of pooled data from 5 vaccine efficacy trials, provided the opportunity to assess HPV prevalence and incidence before pregnancy and after pregnancy resolution. The primary objective was to estimate the HPV prevalence in the same group of women before and after pregnancy. The secondary objective was to evaluate the incidence of HPV infection after pregnancy in women who were HPV-negative at their last visit before pregnancy and who continued with the study postpartum. Lastly, we assessed the effect of potential risk factors such as pregnancy, history of HPV infection, cigarette smoking, number of sexual partners, age, region, and outcome of pregnancy on the incidence of HPV infection.

## METHODS

### Study Design and Participants

This study is a pooled analysis using data from women enrolled in the control arms of 5 AS04-HPV-16/18 vaccine efficacy trials (Supplementary Table 1). The efficacy trials were registered on https://clinicaltrials.gov under numbers NCT001226810 (HPV-008 trial), NCT00294047 (HPV-015 trial), NCT00316693 and NCT00929526 (HPV-032/063 trials), and NCT00779766 (HPV-039 trial). Results of the studies have been published previously [18–23]. Women were recruited from 4 regions (Asia Pacific, Europe, North America, and Latin America) for the HPV-008 and HPV-015 studies [18, 21], in Japan for the HPV-032 and HPV-063 studies [19, 20], and in China for the HPV-039 studies [22, 23].

We defined the following cohorts for the analyses (Supplementary Figure 1): Cohort 1: Nonpregnant cohort—women who did not become pregnant during the trial; Cohort 2: Pregnant cohort—women who became pregnant during the trial or the extended follow up; Cohort 3: Postresolution cohort—women who became pregnant during the trial or the extended follow up and had at least 1 cervical sample collected after pregnancy; Cohort 4: Pregnant HPV-negative cohort—women who became pregnant during the trial or the extended follow up, were HPV negative at the last visit before pregnancy, and had at least 1 cervical sample collected after pregnancy; Cohort 5: Nonpregnant HPV-negative cohort—nonpregnant women who were HPV negative at the time of the last dose of control vaccine administration and had at least 1 cervical sample collected after the last dose of control administration. We computed pre- and postpregnancy HPV prevalence rates in the pregnant and postresolution cohorts, respectively, and the HPV incidence rates in the pregnant HPV-negative and nonpregnant HPV-negative cohorts.

Ethical approval from independent institutional review boards was obtained for all the clinical trials included in this work, and each woman provided written informed consent before the performance of any study-specific procedure. All studies followed the Declaration of Helsinki. Only deidentified data were used in the pooled analysis.

### Procedures

Included women were enrolled previously in 1 of the clinical trials (HPV-008, HPV-015, HPV-032/HPV-063, and HPV-039), regardless of their cytological, serological, and HPV DNA status, where they were randomized to the control arm of the study and received a hepatitis A vaccine (HPV-008 and HPV-032/HPV-063) or an aluminum hydroxide (AlOH_3_) placebo (HPV-015 and HPV-039).

Cervical sample collection, HPV DNA testing, gynecological and cytopathological examinations, and testing of cervical samples for the presence of DNA from 14 hr-HPV strains (16, 18, 31, 33, 35, 39, 45, 51, 52, 56, 58, 59, 66, and 68) using broad-spectrum polymerase chain reaction (PCR), and type-specific PCR for detecting HPV-16 and HPV-18, were performed as described previously [24–26]. No cervical samples were collected from pregnant women in any of the 5 trials.

The 5 trials were sponsored by GSK, allowing authors to extract data from the database owned by the company.

### Statistical Analyses

We analyzed demographic and baseline characteristics with descriptive statistics. The primary objective of our analysis was to estimate HPV prevalence in cohorts 2 and 3, that is, before and after pregnancy. We determined HPV prevalence at the women’s last visit before pregnancy and at the first visit after pregnancy for 5 groups of HPV types—any HPV, any hr-HPV, HPV-16/18, HPV-16, and HPV-18. We calculated the incidence of HPV infection after pregnancy in cohort 4 (pregnant HPV-negative women) overall and stratified by the duration between pregnancy resolution and the first postresolution visit (0–3, 3–6, or >6 months), age at pregnancy resolution, region, and outcome of pregnancy.

We used the Cox proportional hazards model to analyze the potential risk factors for HPV infection in cohorts 4 and 5. Potential risk factors included the following: age at start of follow-up, history of previous HPV infection, pregnancy and its outcome, duration of follow-up, and region. Analysis was performed separately on the pooled HPV-008 and HPV-015 studies and the pooled HPV-032/063 and HPV-039 studies because these 2 groups of studies differed by the availability of the study entry questionnaire data, targeted age groups, and the assays used for HPV detection. A behavioral questionnaire was self-completed only for 2 studies (HPV-008 and HPV-015), providing information about smoking status and number of sexual partners. These variables were collected and assessed as potential risk factors in the pooled HPV-008 and HPV-015 studies. We used SAS 9.2 (SAS Institute, Cary, North Carolina, USA) for all statistical analyses.

## RESULTS

### Participants

A total of 15 754 women were enrolled in the control arm of the 5 efficacy studies (Supplementary Figure 1). Of those 3001 (19.0%) reported at least 1 pregnancy during the studies (Table 1). Of these pregnant women, 2556 (85.2%) continued with the studies after pregnancy. Among them, 2448 (95.8%) women had cervical samples collected at some point after pregnancy (Supplementary Table 1).

**Table 1.** Baseline Characteristics of Control Arm Subjects Included in the Analysis

Characteristics		Pregnant Cohort	Nonpregnant Cohort
All trials	Parameters	N = 3001	N = 12 753
Age (year) at the first dose of control vaccine administration, mean ± SD		22.5 ± 4.0	24.1 ± 8.1
		n (%)	n (%)
Region/country	Europe	334 (11.1%)	3459 (27.1%)
	North America	386 (12.9%)	1903 (14.9%)
	Latin America	442 (14.7%)	1682 (13.2%)
	Asia-Pacific	1839 (61.3%)	5709 (44.8%)
History of HPV infection	Yes	1200 (40.0%)	3630 (28.5%)
	No	1662 (55.4%)	9104 (71.4%)
	Missing	139 (4.6%)	19 (0.1%)
Age at pregnancy resolution (pregnant cohort) or at last dose of control vaccination (nonpregnant cohort), in years	15–17	41 (1.4%)	2242 (17.6%)
	18–20	331 (11.0%)	1955 (15.3%)
	21–24	903 (30.1%)	4192 (32.9%)
	25–34	1571 (52.3%)	2649 (20.8%)
	35–44	93 (3.1%)	1245 (9.8%)
	≥45	2 (0.1%)	470 (3.7%)
	Missing	60 (2.0%)	0 (0.0%)
Pregnancy outcome	Vaginal delivery	1516 (50.5%)	-
	Caesarean section	750 (25.0%)	-
	Elective abortion	387 (12.9%)	-
	Miscarriage	241 (8.0%)	-
	Others	75 (2.5%)	-
	Missing	32 (1.1%)	-
Duration from pregnancy resolution to first postresolution cervical sample collection (pregnant cohort) or from last dose vaccination to first sample collection post vaccination (nonpregnant cohort)	<3 months	332 (11.1%)	228 (1.8%)
	3–6 months	1426 (47.5%)	6060 (47.5%)
	>6 months	690 (23.0%)	5540 (43.4%)
	Missing	553 (18.4%)	925 (7.3%)
**HPV-008/015 trials**		N = 2158	N = 10 049
Cigarette smoking at enrollment	<0.5 pack-years	1731 (80.2%)	8040 (80.0%)
	≥0.5 pack-years	412 (19.1%)	1798 (17.9%)
	missing	15 (0.7%)	211 (2.1%)
Number of sexual partners at enrollment	0	193 (8.9%)	2034 (20.2%)
	1	1176 (54.5%)	4055 (40.4%)
	2–5	650 (30.1%)	2978 (29.6%)
	>5	118 (5.5%)	761 (7.6%)
	missing	21 (1.0%)	221 (2.2%)

Abbreviations: HPV, human papillomavirus; N, total number of subjects in each group; n, number of subjects per category; SD, standard deviation.

### HPV Prevalence Before and After Pregnancy

In the HPV prevalence analyses, 2862 pregnant women were included in cohort 2; of them, 2448 (in cohort 3) also could be analyzed after pregnancy (Table 2). The overall HPV prevalence before and after pregnancy was similar (25.4% and 24.3%, respectively). Similar prevalence before and after pregnancy also was detected for any hr-HPV (20.8 % and 19.8 %), HPV-16 (4.2% and 3.7%), and HPV-18 (2.0% and 1.8%) types (Table 2). In the postresolution cohort (cohort 3), HPV prevalence was higher in women 15–17 years old than in other age groups for all 5 HPV groups. In this same cohort, HPV prevalence was lowest among subjects from China.

**Table 2.** Human Papillomavirus Prevalence Before Pregnancy and Post Pregnancy Resolution (Pregnant Cohort, N = 3001)

		Prevalence				
		Any HPV	hr-HPV	HPV16/18	HPV16	HPV18
	N	% (95% CI)	% (95% CI)	% (95% CI)	% (95% CI)	% (95% CI)
Prepregnancy	2862	25.4 (23.8–27.0)	20.8 (19.3–22.3)	5.9 (5.1–6.8)	4.2 (3.5–5.0)	2.0 (1.5–2.6)
Postresolution	2448	24.3 (22.6–26.0)	19.8 (18.2–21.4)	5.4 (4.5–6.3)	3.7 (3.0–4.5)	1.8 (1.3–2.4)
By age at resolution:						
15–17 years	36	47.2 (30.4–64.5)	38.9 (23.1–56.5)	19.4 (8.2–36.0)	13.9 (4.7–29.5)	5.6 (0.7–18.7)
18–20 years	298	42.3 (36.6–48.1)	37.2 (31.7–43.0)	13.1 (9.5–17.4)	9.1 (6.1–12.9)	4.7 (2.6–7.8)
21–24 years	756	26.6 (23.5–29.9)	21.8 (18.9–24.9)	6.8 (5.1–8.8)	4.9 (3.5–6.7)	2.0 (1.1–3.2)
25–34 years	1268	18.4 (16.3–20.6)	14.5 (12.6–16.6)	2.7 (1.9–3.7)	1.7 (1.0–2.5)	1.0 (0.6–1.8)
35–44 years	88	19.3 (11.7–29.1)	11.4 (5.6–19.9)	0.0 (0.0–4.1)	0.0 (0.0–4.1)	0.0 (0.0–4.1)
≥45 years	2	0.0 (0.0–84.2)	0.0 (0.0–84.2)	0.0 (0.0–84.2)	0.0 (0.0–84.2)	0.0 (0.0–84.2)
by region:						
Asia-Pacific^a^	899	19.5 (16.9–22.2)	16.4 (14.0–18.3)	3.9 (2.7–5.4)	2.1 (1.3–3.3)	1.8 (1.0–2.9)
Europe	257	33.8 (28.1–40.0)	28.8 (23.3–34.8)	11.7 (8.0–16.2)	8.2 (5.1–12.2)	4.3 (2.2–7.5)
Latin America	355	32.7 (27.8–37.8)	26.2 (21.7–31.1)	6.8 (4.4–9.9)	4.5 (2.6–7.2)	2.2 (1.0–4.4)
North America	277	39.4 (33.6–45.4)	30.0 (24.6–35.7)	9.4 (6.2–13.4)	7.6 (4.8–11.4)	2.2 (0.8–4.6)
China	660	16.2 (13.5–19.2)	13.2 (10.7–16.0)	2.4 (1.4–3.9)	2.0 (1.0–3.3)	0.4 (0.1–1.3)

Abbreviations: CI, confidence interval; HPV, human papillomavirus; hr-HPV, high-risk HPV; N, number of participants in each group.

^a^Excluding China.

Most subjects (73.1% in the 2 multi-country studies, 82.1% in the studies in China and Japan; Supplementary Table 2) were HPV negative at first visit after pregnancy.

### Incidence of hr-HPV Infection is Highest Between 3–6 Months After Pregnancy

Of the 1733 women who were HPV negative at the last visit before pregnancy (cohort 4), 357 cases of hr-HPV infection (incidence of 108.4 per 1000 person-years), including 99 cases of infection with HPV-16 or HPV-18 (27.3 per 1000 person-years), were detected (Table 3). The incidence of infection with any hr-HPV type(s) was 40.1 per 1000 person-years within the first 3 months post pregnancy. The incidence increased to 266.7 per 1000 person-years in the next 3 months then decreased to 95.7 per 1000 person-years after 6 months. The same trend of highest incidence in the second 3-month period after pregnancy was observed for HPV-16/18 and HPV-18, but not for HPV-16 (Supplementary Figure 2). Incidence rates postpregnancy resolution showed the same trend for all 5 groups of HPV in individual trials (Supplementary Table 3). In the 2 main contributing studies, that is, HPV-008 and HPV-039, the incidence of infection with hr-HPV increased from 50.0 per 1000 and 15.7 per 1000 person-years within the first 3 months after pregnancy to 360.8 per 1000 and 139.6 per 1000 person-years in the next 3 months post pregnancy, respectively. Incidence rates then decreased to 146.1 per 1000 and 66.0 per 1000 person-years after 6 months (Supplementary Table 3).

**Table 3.** Incidence of Human Papillomavirus Infection (n/1000 Person-Years) With Respect to Time After Pregnancy Resolution (Pregnant HPV-Negative Cohort, N = 1733)

	Duration Between the Pregnancy Resolution and the First HPV Detection Post Resolution of Pregnancy											
	<3 months			3–6 months			>6 months			Any		
HPV type (s)	Total person years	n	Incidence^a^ (95% CI)	Total person years	n	Incidence^a^ (95% CI)	Total person years	n	Incidence^a^ (95% CI)	Total person years	n	Incidence^a^ (95% CI)
Any HPV	423.8	21	49.6 (30.7–75.8)	379.0	122	321.9 (267.3–384.4)	2378.7	274	115.2 (102.0–129.7)	3181.5	417	131.1 (118.8–144.3)
Any hr-HPV	424.0	17	40.1 (23.4–64.2)	382.5	102	266.7 (217.4–323.7)	2486.6	238	95.7 (83.9–108.7)	3293.1	357	108.4 (97.5–120.3)
HPV 16	425.3	3	7.1 (1.5–20.6)	398.3	6	15.1 (5.5–32.8)	2845.1	49	17.2 (12.7–22.8)	3668.8	58	15.8 (12.0–20.4)
HPV 18	425.5	0	0.0 (0.0–8.7)	398.5	12	30.1 (15.6–52.6)	2870.5	35	12.2 (8.5–17.0)	3694.5	47	12.7 (9.4–16.9)
HPV 16/18	425.3	3	7.1 (1.5–20.6)	397.0	18	45.3 (26.9–71.7)	2802.0	78	27.8 (22.0–34.7)	3624.3	99	27.3 (22.2–33.3)

Abbreviations: CI, confidence interval; HPV, human papillomavirus; hr-HPV, high-risk HPV; n, number of participants with this event.

^a^per 1000 person-years.

### Risk Factors for Acquiring hr-HPV Infection in the HPV-Negative Cohorts

Potential risk factors for HPV infection were analyzed with the Cox proportional hazards model in the pregnant HPV-negative women (cohort 4) and in the nonpregnant HPV-negative women (cohort 5).

In pregnant HPV-negative women from the HPV-008 and HPV-015 trials (Table 4), incidence of hr-HPV infection decreases progressively with increasing age at pregnancy resolution. This factor varied from a hazard ratio (HR) of 0.4 (95% confidence interval [CI], 0.2–0.9) for women of 25–34 years old to an HR of 0.2 (95% CI, 0.1–0.6) for women of 35 years or older compared to women of 15–17 years old. Elective abortion (not including spontaneous miscarriage) as the outcome of pregnancy was associated with a higher risk of hr-HPV infection postresolution in comparison to vaginal delivery (HR, 2.0; 95% CI, 1.4–2.8). Miscarriage, however, was not identified as a significant risk factor for hr-HPV infection when compared to vaginal delivery (HR, 0.8; 95% CI, 0.5–1.2). A history of previous infection with hr-HPV type also more than doubled the risk of hr-HPV infection (HR, 2.7; 95% CI, 2.0–3.6). In the nonpregnant cohort from the HPV-008 and HPV-015 trials, the risk factors were age, previous HPV infections, smoking, and number of sexual partners. The risk also was somewhat different among the various regions (Supplementary Table 4).

**Table 4.** Risk Factor Analysis of the Cox Proportional Hazards Model for Time to Any High Risk-HPV Infection (Pregnant HPV-Negative Cohort, N = 1733)

						95% CI	
Risk Factor	Categories	N	n	*P* value	HR	LL	UL
**HPV-008/015 trials**							
Age groups (years) at day 0 of follow-up	15–17 years	16	8	—	—	—	—
	18–20 years	161	62	.858	0.9	0.4	2.0
	21–24 years	366	85	.172	0.6	0.3	1.3
	25–34 years	576	97	.020	0.4	0.2	0.9
	≥35 years^a^	69	14	.003	0.2	0.1	0.6
Region	Europe	150	39	—	—	—	—
	North America	162	45	.990	1.0	0.6	1.6
	Latin America	225	59	.614	1.1	0.7	1.8
	Asia Pacific	651	123	.672	0.9	0.6	1.4
Method of resolution of pregnancy	Vaginal delivery	698	129	—	—	—	—
	Caesarean section	229	50	.600	1.1	0.8	1.6
	Elective abortion	111	52	<.001	2.0	1.4	2.8
	Miscarriage	131	29	.295	0.8	0.5	1.2
	Others	8	1	.852	0.8	0.1	6.0
Duration since end of pregnancy to the time of the first post resolution sample collection	<3 months	165	36	—	—	—	—
	3–6 months	678	161	.415	1.2	0.8	1.7
	>6 months	345	69	.698	1.1	0.7	1.7
History of HPV infection	No any hr-HPV positive result	958	185	—	—	—	—
	Previous any hr-HPV positive result	230	81	<.001	2.7	2.0	3.6
Smoking status at enrollment (pack-years)	<0.5 pack-years	1002	217	.	.	.	.
	≥0.5 pack-years	183	49	.590	0.9	0.6	1.3
Number of sexual partners at enrollment	0	103	32	—	—	—	—
	1	718	139	.314	0.8	0.5	1.2
	2–5	306	86	.905	1.0	0.6	1.6
	>5	54	9	.042	0.4	0.2	1.0
**HPV-032/063/039 trials**							
Age groups (years) at day 0 of follow-up^b^	18–20 years	10	4	—	—	—	—
	21–24 years	131	25	.079	0.4	0.1	1.1
	25–34 years	404	62	.065	0.4	0.1	1.1
Region	Asia Pacific^c^	26	8	—	—	—	—
	China	519	83	.006	0.3	0.2	0.7
Method of resolution of pregnancy	Vaginal delivery	200	37	—	—	—	—
	Caesarean section	221	28	.450	0.8	0.5	1.4
	Elective abortion	108	25	.992	1.0	0.6	1.7
	Miscarriage	9	0	.978	0.0	0.0	—
	Others	7	1	.405	0.4	0.1	3.2
Duration since end of pregnancy to the time of the first postresolution sample collection	<3 months	40	10	—	—	—	—
	3–6 months	341	52	.444	0.8	0.4	1.5
	>6 months	164	29	.620	0.8	0.4	1.8
History of HPV infection	No any hr-HPV positive result	454	59	—	—	—	—
	Previous any hr-HPV positive result	91	32	<.001	3.4	2.2	5.4

Abbreviations: CI, confidence interval; HPV, human papillomavirus; hr-HPV, high-risk HPV; HR, hazard ratio; LL, lower limit of confidence interval; N, total number of subjects in the category; n, number of subjects with hr-HPV infection status in the category; UL, upper limit of confidence interval.

^a^The age groups 35–44 years and ≥45 years are pooled together as the number of subjects in ≥45 years were very low.

^b^There were no subjects in the 35–44 years and ≥45 years age groups in the pooled HPV032/069 and HPV-039 studies.

^c^Excluding China.

In the HPV-032/063 and HPV-039 trials where information about sexual behavior was not captured, history of previous hr-HPV infection was associated with higher risk of acquiring infection with hr-HPV (HR, 3.4; 95% CI, 2.2–5.4) in pregnant HPV-negative women. Risk of infection by HPV was lower in China than in Japan (HR, 0.3; 95% CI, 0.2–0.7; Table 4). The same risk factors were observed in the nonpregnant cohort (Supplementary Table 4).

The association of pregnancy with hr-HPV infection also was analyzed in the combined population of the 2 HPV-negative cohorts (combined cohorts 4 and 5; Table 5). In women from the HPV-008 and HPV-015 trials (N = 8092), pregnancy was associated with a small increased risk for hr-HPV infection (HR, 1.2; 95% CI, 1.0–1.4). This, however, was not observed in women (N = 2451) from the HPV-032/063 and HPV-039 trials (HR, 0.9; 95% CI, 0.7–1.1).

**Table 5.** Pregnancy as Risk Factor Analysis of the Cox Proportional Hazards Model for Time to Any High Risk-HPV Infection (HPV-Negative Cohort)

						95% CI	
Risk factor	Categories	N	n	*P* value	HR	LL	UL
**HPV-008/015 trials**							
Pregnancy status	Nonpregnant	6904	2552	—	—	—	—
	Pregnant	1188	266	.008	1.2	1.0	1.4
**HPV-032/063/039 trials**							
Pregnancy status	Nonpregnant	1906	588	—	—	—	—
	Pregnant	545	91	.235	0.9	0.7	1.1

Abbreviations: CI, confidence interval; HPV, human papillomavirus; HR, hazard ratio; LL, lower limit of confidence interval; N, total number of subjects in the category; n, number of subjects with high risk-HPV infection status in the category; UL, upper limit of confidence interval.

Supplementary Figure 3 elaborates on the clinical relevance of the study findings in a form that could be shared with patients by healthcare professionals.

## DISCUSSION

We examined HPV prevalence in women before and after pregnancy from the control arms of 5 clinical trials of the HPV-16/18 AS04-adjuvanted vaccine. These women, who were enrolled regardless of their cytological, serological, and HPV DNA status and who were not vaccinated against HPV infection, therefore approximate the general population. We also examined HPV incidence after pregnancy in women that were HPV negative before pregnancy. These analyses were insightful about the risks of HPV infection following pregnancy. Finally, we explored several risk factors for acquiring HPV infection potentially during or shortly after pregnancy.

When comparing prevalence in women before and after pregnancy, we found no meaningful differences in the overall prevalence of hr-HPV or HPV-16/18 infections. Pregnant women, in general, have fewer new sexual partners than nonpregnant women, and as the number of sexual partners is a known risk factor for HPV infection [5], they are less likely to acquire new infections. The transient nature of many HPV infections also may explain partly this finding [4]. As cervical samples during pregnancy were not available, we were not able to validate the suggestion that women had a lower HPV clearance during pregnancy with a catch up postpartum [27]. Previous studies in pregnant versus nonpregnant women found conflicting results of HPV prevalence. Some reported it was higher in pregnant women [10, 11], while others reported that there was no difference in HPV prevalence [12, 13]. In those studies, however, the HPV prevalence was not assessed in the women before pregnancy, so their HPV prevalence at the start is unknown [11, 28]. The outcomes, therefore, are debatable. A study assessing HPV prevalence before pregnancy, at various trimesters during pregnancy, and at several timepoints after pregnancy may shed light on the dynamics of HPV infection and clearance around pregnancy.

In our study, most women, either before or after pregnancy, were HPV negative. These data are similar to those found for the general female population [17]. These women are at risk of acquiring HPV infection and would benefit from preventive measures to reduce their risk of infection and of further developing HPV-associated cervical cancer.

Few studies reported the incidence of HPV infection after pregnancy, but there were no data on the risk factors of HPV infection after pregnancy. When analyzing women who were HPV negative before pregnancy, we found that the incidence of HPV infection for any HPV, any hr-HPV, HPV-16, HPV-18, and HPV-16/18, was the highest 3 to 6 months after resolution of pregnancy. The incidence increased drastically after the first 3 months postresolution then declined after the second 3-month period. A recent review of the literature on sexual behavior during and after pregnancy found that the average time of resumption of intercourse was 6 to 8 weeks postpartum, although the initial frequency is very low. The frequency increases at 3 months, 4 to 6 months, and at 1 year after pregnancy, but even then it does not reach prepregnancy rates [29]. The data on resumption and frequency of intercourse may explain the peak seen in increased HPV incidence at 3 to 6 months after pregnancy as this period is close to the typical time of 6 to 8 weeks needed for the healing of the cervix after pregnancy. In addition, other postpartum sexual behaviors may impact the incidence of HPV infection after pregnancy. The number of sexual partners of the women or the exposure of their partner to other sexual partners outside of the relationship could be potential factors to consider, as postpartum depression and alteration of sexual function can alter the relationship [30]. Among other potential factors, it is noteworthy that a recent study reported about half of the women who had resumed sexual intercourses 6 weeks after birth were not using contraception, including condoms [31].

The incidence of any hr-HPV infection in women after pregnancy was 108.4 per 1000 person-years, which is much lower than the incidence of 217.9 per 1000 person-years reported in women who had recently commenced sexual activity in the control arm of the HPV-008 trial [32]. The women who experienced first sexual activity in the HPV-008 study had a mean age of 17.4 +/- 2.1 years [32], while in our study the mean age at pregnancy resolution was 25.1 +/- 4.3 years. Incidence of HPV stratified by age groups, however, shows that the youngest women (15–24 years old) endure the highest incidence of HPV infections (Supplementary Table 5), underlining the reduced risk of infection at an increased age that we found.

Our exploratory analysis of risk factors found younger age at pregnancy resolution (reversely associated), history of previous HPV infection, elective abortion, and the number of sexual partners were risk factors for postresolution HPV infection. Smoking increased the risk of acquiring HPV infection in the nonpregnant women, though this was not observed in the pregnant cohort. The risk also varied among regions, being the highest in the Americas and lowest in Asia. Analysis using the Cox proportional hazards model did not confirm pregnancy as a risk factor in our study population.

The most significant risk factor for any hr-HPV infection was a previous infection with HPV (HR, 2–4). This risk factor may be explained by the other mentioned lifestyle factors (such as smoking, number of sexual partners, etc.), genetic susceptibility to HPV infection, or dormant HPV infection that was not cleared and remained in basal layer of the cervix. Such dormant HPV infection could be reactivated by pregnancy or delivery and be detected as new infection.

There are several limitations to the current study. First, there were no cervical samples collected during pregnancy. The value of taking such samples would be difficult to justify due to the potential risk of the procedure during pregnancy. Therefore, we were unable to assess the prevalence, incidence, and clearance of HPV infection during pregnancy. It would be valuable to determine these parameters during the pregnancy versus before and after pregnancy. For instance, a lower HPV prevalence during pregnancy could be due to a lower infection rate if fewer sexual partners are encountered. Second, initially HPV-negative women with their first postresolution HPV-positive cervical sample could have been infected any time between the last test before pregnancy and the first postpartum test. They were all considered as incident cases with the infection occurring after pregnancy, even though sexual activity usually continues, but at a lower frequency, during the first 2 trimesters of pregnancy [30, 33]. In that way, the postresolution incidence potentially could be overestimated. Interestingly, when the first postpartum test was made between 3 to 6 months after pregnancy, the measured incidence was higher and might correlate with the increased sexual activity that is known to occur at that time. Still, relatively few women had their first postresolution cervical samples collected within the first 3 months after pregnancy. The incidence during the 3–6 months postresolution period thus could be overestimated if some of the incident cases who acquired HPV infection before this period only were detected then. Smoking status was recorded at study enrollment, though women may have quit smoking at pregnancy detection, as advocated by their physician. This information was not captured and may have impacted the results, but is unlikely to change the conclusion of the analysis. Finally, our analysis had to be stratified due to differences in data availability between the HPV-008/015 and the HPV-032/063/039 studies. We used the available data from the control arms of studies that differed in population, design, and objectives. This heterogeneity reduced the sample size and the opportunity to confirm some potential risk factors of HPV infection.

The results of this pooled analysis add to the current understanding about HPV prevalence and incidence around pregnancy. Prevalence of HPV was similar before and after pregnancy, suggesting that despite an altered immune system, the risk of infection by HPV may not change significantly during pregnancy (although we had no direct access to this information). Another finding was that HPV incidence in previously HPV-negative women was the highest in the second 3-month period after pregnancy. This may reflect the timing of resumption and frequency of sexual activities.

## Supplementary Data

Supplementary materials are available at *Open Forum Infectious Diseases* online. Consisting of data provided by the authors to benefit the reader, the posted materials are not copyedited and are the sole responsibility of the authors, so questions or comments should be addressed to the corresponding author.

ofz486_suppl_Supplementary_Material
