# Erratum to: Prevalence and Incidence of Human Papillomavirus (HPV) Infection Before and After Pregnancy: Pooled Analysis of the Control Arms of Efficacy Trials of HPV-16/18 AS04-Adjuvanted Vaccine

**DOI:** 10.1093/ofid/ofaa036

**Published:** 2020-02-28

**Authors:** Jing Chen, Kusuma Gopala, Akarsh Puthatta, Frank Struyf, Dominique Rosillon

*Open Forum Infectious Diseases*, Volume 6, Issue 12, December 2019, ofz486, https://doi.org/10.1093/ofid/ofz486

**Published:** 04 December 2019

An error appeared in an article published in the December 2019 issue of the journal, [Prevalence and Incidence of Human Papillomavirus (HPV) Infection Before and After Pregnancy: Pooled Analysis of the Control Arms of Efficacy Trials of HPV-16/18 AS04-Adjuvanted Vaccine, *Open Forum Infectious Diseases, *Volume 6, Issue 12, December 2019, ofz486]. The author listing of “P K Akarsh” should state “Akarsh Puthatta”, with Akarsh being first name and Puthatta family name. This has now been corrected online.

