# Supplementary material for: Prevalence and Incidence of Human Papillomavirus (HPV) Infection Before and After Pregnancy: Pooled Analysis of the Control Arms of Efficacy Trials of HPV-16/18 AS04-Adjuvanted Vaccine
Source: Open Forum Infect Dis. 2019 Dec 4;6(12):ofz486. doi: 10.1093/ofid/ofz486 (PMC6892569; doi:10.1093/ofid/ofz486)

**Supplemental Table 1 Number of subjects potentially eligible for the analysis in the current study**

| **HPV-16/18 AS04-adjuvanted vaccine  clinical trial (reference)** | **Number of subjects in the control arm, n** | | | | | | |
| --- | --- | --- | --- | --- | --- | --- | --- |
|  | **TVC (Control arm)** | **With at least one pregnancy recorded during the study period** | **Returned for at least 1 visit post resolution of first pregnancy** | **With cervical sample collected post resolution of first pregnancy** | | | |
|  |  |  |  | **All** | **Within 3 months of resolution** | **Between 3-6 months of resolution** | **After 6 months post resolution** |
| **HPV-008** [1] | 9,336 | 1,857 | 1,528 | 1,463 | 236 | 823 | 404 |
| **HPV-015** [2] | 2,871 | 301 | 273 | 269 | 43 | 150 | 76 |
| **HPV-032** [3]**/ HPV-063** [4] | 521 | 71 | 60 | 56 | 3 | 34 | 19 |
| **HPV-039** [5] | 3,026 | 772 | 695 | 660 | 50 | 419 | 191 |
| **Total** | 15,754 | 3,001 | 2,556 | 2,448 | 332 | 1,426 | 690 |

HPV, human papillomavirus; TVC, total vaccinated cohort; n, number of participants in this group.

[1] Apter D, Wheeler CM, Paavonen J, et al. Efficacy of Human Papillomavirus 16 and 18 (HPV-16/18) AS04-Adjuvanted Vaccine against Cervical Infection and Precancer in Young Women: Final Event-Driven Analysis of the Randomized, Double-Blind PATRICIA Trial. Clin Vaccine Immunol **2015**; 22:361-73.

[2] Skinner SR, Szarewski A, Romanowski B, et al. Efficacy, safety, and immunogenicity of the human papillomavirus 16/18 AS04-adjuvanted vaccine in women older than 25 years: 4-year interim follow-up of the phase 3, double-blind, randomised controlled VIVIANE study. The Lancet **2014**; 384:2213-27.

[3] Konno R, Tamura S, Dobbelaere K, Yoshikawa H. Efficacy of human papillomavirus type 16/18 AS04-adjuvanted vaccine in Japanese women aged 20 to 25 years: final analysis of a phase 2 double-blind, randomized controlled trial. Int J Gynecol Cancer **2010**; 20:847-55.

[4] Konno R, Yoshikawa H, Okutani M, et al. Efficacy of the human papillomavirus (HPV)-16/18 AS04-adjuvanted vaccine against cervical intraepithelial neoplasia and cervical infection in young Japanese women. Hum Vaccin Immunother **2014**; 10:1781-94.

[5] Zhu F-C, Chen W, Hu Y-M, et al. Efficacy, immunogenicity and safety of the HPV-16/18 AS04-adjuvanted vaccine in healthy Chinese women aged 18–25 years: Results from a randomized controlled trial. Int J Cancer **2014**; 135:2612-22.

Supplemental Table 2 HPV prevalence before pregnancy and post pregnancy resolution by combined HPV-008/015 and HPV-032/063/039 trials (Pregnant cohort, N=3,001)

|  |  |  | **Prevalence** | | | | |
| --- | --- | --- | --- | --- | --- | --- | --- |
|  |  |  | **Any HPV** | **hr-HPV** | **HPV-16/18** | **HPV-16** | **HPV-18** |
| **Clinical Trials** |  | N | % (95% CI) | % (95% CI) | % (95% CI) | % (95% CI) | % (95% CI) |
| HPV-008/015 | Pre-pregnancy | 2,035 | 27.7 (25.8-29.7) | 22.5 (20.7-24.4) | 6.9 (5.8-8.1) | 4.8 (3.9-5.8) | 2.5 (1.9-3.3) |
|  | Post-resolution, overall | 1,732 | 26.9 (24.8-29.1) | 21.8 (19.9-23.8) | 6.3 (5.2-7.5) | 4.3 (3.4-5.3) | 2.2 (1.6-3.0) |
|  | by age at resolution: |  |  |  |  |  |  |
|  | 15-17 years | 36 | 47.2 (30.4-64.5) | 38.9 (23.1-56.5) | 19.4 (8.2-36.0) | 13.9 (4.7-29.5) | 5.6 (0.7-18.7) |
|  | 18-20 year | 284 | 43.0 (37.1-48.9) | 37.7 (32.0-43.6) | 13.4 (9.6-17.9) | 9.2 (6.1-13.1) | 4.9 (2.7-8.1) |
|  | 21-24 years | 570 | 28.6 (24.9-32.5) | 23.2 (19.8-26.8) | 7.2 (5.2-9.6) | 5.3 (3.6-7.4) | 2.1 (1.1-3.6) |
|  | 25-34 years | 752 | 19.6 (16.8-22.6) | 15.3 (12.8-18.1) | 3.1 (2.0-4.6) | 1.7 (0.9-2.9) | 1.3 (0.6-2.4) |
|  | 35-44 years | 88 | 19.3 (11.7-29.1) | 11.4 (5.6-19.9) | 0.0 (0.0-4.1) | 0.0 (0.0-4.1) | 0.0 (0.0-4.1) |
|  | ≥45 years | 2 | 0.0 (0.0-84.2) | 0.0 (0.0-84.2) | 0.0 (0.0-84.2) | 0.0 (0.0-84.2) | 0.0 (0.0-84.2) |
|  | by region: |  |  |  |  |  |  |
|  | Asia-pacific | 843 | 18.3 (15.7-21.0) | 15.2 (12.8-17.8) | 3.4 (2.3-4.9) | 1.9 (1.1-3.1) | 1.5 (0.8-2.6) |
|  | Europe | 257 | 33.8 (28.1-40.0) | 28.8 (23.3-34.8) | 11.7 (8.0-16.2) | 8.2 (5.1-12.2) | 4.3 (2.2-7.5) |
|  | Latin America | 355 | 32.7 (27.8-37.8) | 26.2 (21.7-31.1) | 6.8 (4.4-9.9) | 4.5 (2.6-7.2) | 2.2 (1.0-4.4) |
|  | North America | 277 | 39.4 (33.6-45.4) | 30.0 (24.6-35.7) | 9.4 (6.2-13.4) | 7.6 (4.8-11.4) | 2.2 (0.8-4.6) |
| HPV-032/063/039 | Pre-pregnancy | 827 | 19.7 (17.0-22.6) | 16.6 (14.1-19.3) | 3.5 (2.4-5.0) | 2.7 (1.7-4.0) | 0.8 (0.3-1.7) |
|  | Post-resolution, overall | 716 | 17.9 (15.1-20.9) | 14.8 (12.3-17.6) | 3.1 (1.9-4.6) | 2.2 (1.3-3.6) | 0.8 (0.3-1.8) |
|  | by age at resolution: |  |  |  |  |  |  |
|  | 15-17 years | 0 | - | - | - | - | - |
|  | 18-20 years | 14 | 28.6 (8.4-58.1) | 28.6 (8.4-58.1) | 7.1 (0.2-33.9) | 7.1 (0.2-33.9) | 0.0 (0.0-23.2) |
|  | 21-24 years | 186 | 20.4 (14.9-27.0) | 17.7 (12.5-24.0) | 5.4 (2.6-9.7) | 3.8 (1.5-7.6) | 1.6 (0.3-4.6) |
|  | 25-34 years | 516 | 16.7 (13.6-20.2) | 13.4 (10.6-16.6) | 2.1 (1.1-3.8) | 1.6 (0.7-3.0) | 0.6 (0.1-1.7) |
|  | 35-44 years | 0 | - | - | - | - | - |
|  | ≥45 years | 0 | - | - | - | - | - |
|  | by region: |  |  |  |  |  |  |
|  | China | 660 | 16.2 (13.5-19.2) | 13.2 (10.7-16.0) | 2.4 (1.4-3.9) | 2.0 (1.0-3.3) | 0.4 (0.1-1.3) |
|  | Japan | 56 | 37.5 (24.9-51.4) | 33.9 (21.8-47.8) | 10.7 (4.0-21.9) | 5.4 (1.1-14.9) | 5.4 (1.1-14.9) |

CI, confidence interval; HPV, human papillomavirus; hr-HPV, high-risk HPV; N, number of participants in each group.

Supplemental Table 3 Incidence of HPV infection (n/1,000 person-years) in individual studies, with respect to time after pregnancy resolution (HPV-negative pregnant cohort, N=1,733)

|  | | **Duration between the pregnancy resolution and the first HPV detection post resolution of pregnancy** | | | | | | | | | | | | | | |
| --- | --- | --- | --- | --- | --- | --- | --- | --- | --- | --- | --- | --- | --- | --- | --- | --- |
|  | | **< 3 months** | | | **3 – 6 months** | | | **> 6 months** | | | | | **Any** | | | |
| **Clinical**  **trial** | **HPV type(s)** | **Total person years** | **n** | **Incidence^#^**  **(95% CI)** | **Total person years** | **n** | **Incidence^#^**  **(95% CI)** | **Total person years** | | **n** | **Incidence^#^**  **(95% CI)** | **Total person years** | | **n** | **Incidence^#^**  **(95% CI)** |  |
| HPV-008 | Any HPV | 239.9 | 15 | 62.5 (35.0-103.1) | 205.9 | 88 | 427.4 (342.8-526.5) | | 870.4 | 152 | 174.6 (148.0-204.7) | 1,316.2 | | 255 | 193.7 (170.7-219.0) |  |
|  | Any hr-HPV | 240.0 | 12 | 50.0 (25.8-87.3) | 207.8 | 75 | 360.8 (283.8-452.3) | | 917.2 | 134 | 146.1 (122.4-173.0) | 1,365.1 | | 221 | 161.9 (141.2-184.7) |  |
|  | HPV-16 | 240.9 | 3 | 12.4 (2.6-36.4) | 218.6 | 3 | 13.7 (2.8-40.1) | | 1085.9 | 29 | 26.7 (17.9-38.4) | 1,545.4 | | 35 | 22.6 (15.8-31.5) |  |
|  | HPV-18 | 241.1 | 0 | 0 (0-15.3) | 218.6 | 9 | 41.2 (18.8-78.2) | | 1085.7 | 25 | 23.0 (14.9-34.0) | 1,545.4 | | 34 | 22.0 (15.2-30.7) |  |
|  | HPV-16/18 | 240.9 | 3 | 12.4 (2.6-36.4) | 217.7 | 12 | 55.1 (28.5-96.3) | | 1058.8 | 49 | 46.3 (34.2-61.2) | 1,517.4 | | 64 | 42.2 (32.5-53.9) |  |
| HPV-015 | Any HPV | 49.8 | 4 | 80.4 (21.9-205.7) | 47.7 | 6 | 125.8 (46.2-273.4) | | 554.1 | 43 | 77.6 (56.2-104.5) | 651.6 | | 53 | 81.3 (60.9-106.4) |  |
|  | Any hr-HPV | 49.8 | 3 | 60.2 (12.4-176.1) | 48.1 | 5 | 104.0 (33.8-242.6) | | 585.2 | 37 | 63.2 (44.5-87.2) | 683.0 | | 45 | 65.9 (48.1-88.2) |  |
|  | HPV-16 | 50.2 | 0 | 0 (0-73.6) | 49.7 | 0 | 0 (0-74.2) | | 667.8 | 7 | 10.5 (4.2-21.6) | 767.7 | | 7 | 9.1 (3.7-18.8) |  |
|  | HPV-18 | 50.2 | 0 | 0 (0-73.6) | 49.7 | 0 | 0 (0-74.2) | | 672.6 | 6 | 8.9 (3.3-19.4) | 772.5 | | 6 | 7.8 (2.8-16.9) |  |
|  | HPV-16/18 | 50.2 | 0 | 0 (0-73.6) | 49.7 | 0 | 0 (0-74.2) | | 658.4 | 12 | 18.2 (9.4-31.8) | 758.3 | | 12 | 15.8 (8.2-27.6) |  |
| HPV-032/063 | Any HPV | 6.4 | 0 | 0.0 (0.0-575.5) | 4.8 | 5 | 1,035.2 (336.1-2,415.8) | | 12.9 | 3 | 233.5 (48.2-682.3) | 24.1 | | 8 | 332.1 (143.4-654.4) |  |
|  | Any hr-HPV | 6.4 | 0 | 0.0 (0.0-575.5) | 4.8 | 5 | 1,035.2 (336.1-2,415.8) | | 13.9 | 3 | 215.7 (44.5-630.3) | 25.1 | | 8 | 318.2 (137.4-627.0) |  |
|  | HPV-16 | 6.4 | 0 | 0.0 (0.0-575.5) | 5.6 | 0 | 0.0 (0.0-655.2) | | 18.7 | 1 | 53.4 (1.4-297.3) | 30.8 | | 1 | 32.5 (0.8-181.0) |  |
|  | HPV-18 | 6.4 | 0 | 0.0 (0.0-575.5) | 5.4 | 2 | 370.4 (44.9-1,337.9) | | 16.8 | 1 | 59.5 (1.5-331.5) | 28.6 | | 3 | 104.8 (21.6-306.3) |  |
|  | HPV-16/18 | 6.4 | 0 | 0.0 (0.0-575.5) | 5.4 | 2 | 370.4 (44.9-1,337.9) | | 16.8 | 2 | 119.0 (14.4-429.8) | 28.6 | | 4 | 139.8 (38.1-357.9) |  |
| HPV-039 | Any HPV | 127.7 | 2 | 15.7 (1.9-56.6) | 120.5 | 23 | 190.8 (121.0-286.3) | | 941.4 | 76 | 80.7 (63.6-101.1) | 1,189.6 | | 101 | 84.9 (69.2-103.2) |  |
|  | Any hr-HPV | 127.7 | 2 | 15.7 (1.9-56.6) | 121.8 | 17 | 139.6 (81.3-223.5) | | 970.3 | 64 | 66.0 (50.8-84.2) | 1,219.8 | | 83 | 68.1 (54.2-84.4) |  |
|  | HPV-16 | 127.9 | 0 | 0.0 (0.0-28.8) | 124.3 | 3 | 24.1 (5.0-70.5) | | 1,072.8 | 12 | 11.2 (5.8-19.5) | 1,325.0 | | 15 | 11.3 (6.3-18.7) |  |
|  | HPV-18 | 127.9 | 0 | 0.0 (0.0-28.8) | 124.7 | 1 | 8.0 (0.2-44.7) | | 1,095.3 | 3 | 2.7 (0.6-8.0) | 1,347.9 | | 4 | 3.0 (0.8-7.6) |  |
|  | HPV-16/18 | 127.9 | 0 | 0.0 (0.0-28.8) | 124.1 | 4 | 32.2 (8.8-82.5) | | 1,067.9 | 15 | 14.1 (7.9-23.2) | 1,319.9 | | 19 | 14.4 (8.7-22.5) |  |

n, number of participants with this event; CI, confidence interval; HPV, human papillomavirus; hr-HPV, high-risk HPV; ^#^ per 1,000 person-years.

**Supplemental Table 4 Risk factor analysis of the Cox Proportion Hazard model for time to any high risk-HPV infection (HPV negative non-pregnant cohort, N=8,810)**

| **Risk factor** | **Categories** | **N** | **n** | **P-value** | **HR** | **95% CI** | |
| --- | --- | --- | --- | --- | --- | --- | --- |
|  |  |  |  |  |  | **LL** | **UL** |
| **HPV-008/015 trials** | | | | | | | |
| Age groups (years) at day 0 of follow-up | 15-17 years | 1,650 | 818 | . | . | . | . |
|  | 18-20 years | 1,095 | 504 | 0.008 | 0.8 | 0.7 | 1.0 |
|  | 21-24 years | 1,570 | 542 | 0 | 0.6 | 0.5 | 0.7 |
|  | 25-34 years | 1,249 | 383 | 0 | 0.3 | 0.3 | 0.4 |
|  | 35-44 years | 968 | 239 | 0 | 0.2 | 0.1 | 0.2 |
|  | ≥45 years | 372 | 66 | 0 | 0.1 | 0.1 | 0.2 |
| Region | Europe | 2,540 | 1,134 | . | . | . | . |
|  | North America | 1,051 | 412 | 0.002 | 1.2 | 1.1 | 1.4 |
|  | Latin America | 1,091 | 431 | 0.003 | 1.2 | 1.1 | 1.4 |
|  | Asia Pacific | 2,222 | 575 | 0.001 | 0.8 | 0.7 | 0.9 |
| Duration since end of last dose of control vaccine to the time of the first post resolution sample collection | <3 months | 30 | 8 | . | . | . | . |
|  | 3–6 months | 3,414 | 1,288 | 0.914 | 1.0 | 0.5 | 1.9 |
|  | >6 months | 3,460 | 1,256 | 0.696 | 0.9 | 0.4 | 1.7 |
| History of HPV infection | No any hr-HPV positive result | 6,531 | 2,326 | . | . | . | . |
|  | Previous any hr-HPV positive result | 373 | 226 | 0 | 2.0 | 1.7 | 2.3 |
| Smoking status (number of packs per year) | <0.5 pack year | 5,783 | 2,089 | . | . | . | . |
|  | ≥0.5 pack year | 1,102 | 460 | 0.003 | 1.2 | 1.0 | 1.3 |
| Number of sexual partners | 0 | 1,656 | 654 | . | . | . | . |
|  | 1 | 2,992 | 955 | 0.001 | 1.2 | 1.1 | 1.4 |
|  | 2-5 | 1,774 | 760 | 0 | 1.7 | 1.5 | 2.0 |
|  | >5 | 455 | 177 | 0 | 2.0 | 1.7 | 2.5 |
| **HPV-032/063/039 trials** | | | | | | | |
| Age groups (years) at day 0 of follow-up | 18-20 Years | 103 | 34 | . | . | . | . |
|  | 21-24 Years | 1,185 | 366 | 0.285 | 0.8 | 0.6 | 1.2 |
|  | 25+ Years | 618 | 188 | 0.275 | 0.8 | 0.6 | 1.2 |
| Region | Asia Pacific^ | 249 | 84 | . | . | . | . |
|  | China | 1,657 | 504 | 0 | 0.6 | 0.4 | 0.7 |
| Duration since end of last dose of control vaccine to the time of the first post resolution sample collection | < 6 months | 1,165 | 364 | . | . | . | . |
|  | > 6 months | 741 | 224 | 0.946 | 1.0 | 0.8 | 1.2 |
| History of HPV infection | No any hr-HPV positive result | 1,792 | 526 | . | . | . | . |
|  | Previous any hr-HPV positive result | 114 | 62 | 0 | 2.6 | 2.0 | 3.3 |

CI, confidence interval; HPV, human papillomavirus; hr-HPV, high risk-HPV; HR, hazard ratio; LL, lower limit of confidence interval; N, total number of subjects in the category; n, number of subjects with hr-HPV infection status in the category; UR, upper limit of confidence interval. ^Excluding China.

**Supplemental Table 5** **Incidence of cervical HPV positive infection post-resolution of pregnancy, by age at resolution of pregnancy and** **by time post resolution of the pregnancy (HPV-negative pregnant cohort, N=1,733)**

| **Age at resolution of pregnancy** | | **15-17 years** | **18-20 years** | **21-24 years** | **25-34 years** | **35-44 years** | **45+ years** |  |
| --- | --- | --- | --- | --- | --- | --- | --- | --- |
| **Time post pregnancy resolution** | **HPV type** | **Incidence**  **(95% CI)** | **Incidence**  **(95% CI)** | **Incidence**  **(95% CI)** | **Incidence**  **(95% CI)** | **Incidence**  **(95% CI)** | **Incidence**  **(95% CI)** |  |
|  | | | | | | | | |
| **<3 months** | **Any HPV** | 258.4  (6.5-1439.7) | 144.4  (53.0-314.4) | 41.0  (13.3-95.8) | 33.4  (14.4-65.8) | 60.9  (1.5-339.1) | 0.0 (0.0-7528.3) | |
|  | **Any hr-HPV** | 258.4  (6.5-1439.7) | 96.0  (26.1-245.7) | 32.8  (9.0-84.1) | 29.2  (11.7-60.2) | 60.9  (1.5-339.1) | 0.0 (0.0-7528.3) | |
|  | **HPV-16** | 0.0 (0.0-936.3) | 47.8  (5.8-172.6) | 8.2  (0.2-45.6) | 0.0 (0.0-15.4) | 0.0 (0.0-223.4) | 0.0 (0.0-7528.3) | |
|  | **HPV-18** | 0.0 (0.0-936.3) | 0.0 (0.0-87.8) | 0.0 (0.0-30.2) | 0.0 (0.0-15.4) | 0.0 (0.0-223.4) | 0.0 (0.0-7528.3) | |
|  | **HPV-16/18** | 0.0 (0.0-936.3) | 47.8  (5.8-172.6) | 8.2  (0.2-45.6) | 0.0 (0.0-15.4) | 0.0 (0.0-223.4) | 0.0 (0.0-7528.3) | |
|  | | | | | | | | |
| **3-6 months** | **Any HPV** | 278.6  (7.0-1552.0) | 815.5  (546.2-1171.2) | 376.2  (269.9-510.3) | 228.4  (169.0-302.0) | 126.3  (15.3-456.1) | 0.0 (0.0-7528.3) | |
|  | **Any hr-HPV** | 278.6  (7.0-1552.0) | 747.7  (492.8-1087.9) | 318.7  (222.0-443.2) | 170.8  (120.2-235.4) | 126.3  (15.3-456.1) | 0.0 (0.0-7528.3) | |
|  | **HPV-16** | 0.0 (0.0-936.3) | 50.9  (6.2-183.7) | 26.1  (5.4-76.4) | 4.5  (0.1-25.0) | 0.0 (0.0-224.2) | 0.0 (0.0-7528.3) | |
|  | **HPV-18** | 0.0 (0.0-936.3) | 126.6  (41.1-295.6) | 26.1  (5.4-76.2) | 17.9  (4.9-45.9) | 0.0 (0.0-224.2) | 0.0 (0.0-7528.3) | |
|  | **HPV-16/18** | 0.0 (0.0-936.3) | 180.3  (72.5-371.5) | 52.4  (19.2-114.1) | 22.4  (7.3-52.4) | 0.0 (0.0-224.2) | 0.0 (0.0-7528.3) | |
|  | | | | | | | | |
| **>6 months** | **Any HPV** | 375.3  (150.9-773.3) | 211.2  (147.1-293.7) | 135.4  (108.3-167.2) | 95.5  (79.8-113.4) | 78.5  (43.9-129.5) | 160.0  (4.0-891.5) | |
|  | **Any hr-HPV** | 292.3  (107.2-636.1) | 199.9  (139.2-278.0) | 107.4  (83.9-135.5) | 81.4  (67.2-97.7) | 47.5  (22.8-87.4) | 152.7  (3.9-850.6) | |
|  | **HPV-16** | 62.6  (7.6-226.1) | 34.9  (15.1-68.8) | 19.4  (10.8-32.0) | 15.4  (9.9-22.9) | 0.0 (0.0-15.2) | 0.0 (0.0-389.9) | |
|  | **HPV-18** | 32.7  (0.8-182.4) | 38.6  (17.6-73.3) | 11.4  (5.2-21.6) | 8.9  (4.9-15.0) | 8.3  (1.0-30.0) | 0.0 (0.0-389.9) | |
|  | **HPV-16/18** | 99.9  (20.6-292.0) | 63.7  (34.8-106.8) | 30.1  (19.1-45.2) | 23.4  (16.4-32.4) | 8.3  (1.0-30.0) | 0.0 (0.0-389.9) | |

CI, confidence interval; HPV, human papillomavirus; hr-HPV, high risk-HPV; LL, lower limit of confidence interval; UL, upper limit of confidence interval. Incidence values reported per 1,000 person-years.

**Supplemental Figure 1 Subject disposition flowchart.** TVC: total vaccinated cohort; N: number of subjects; HPV: human papillomavirus.

**
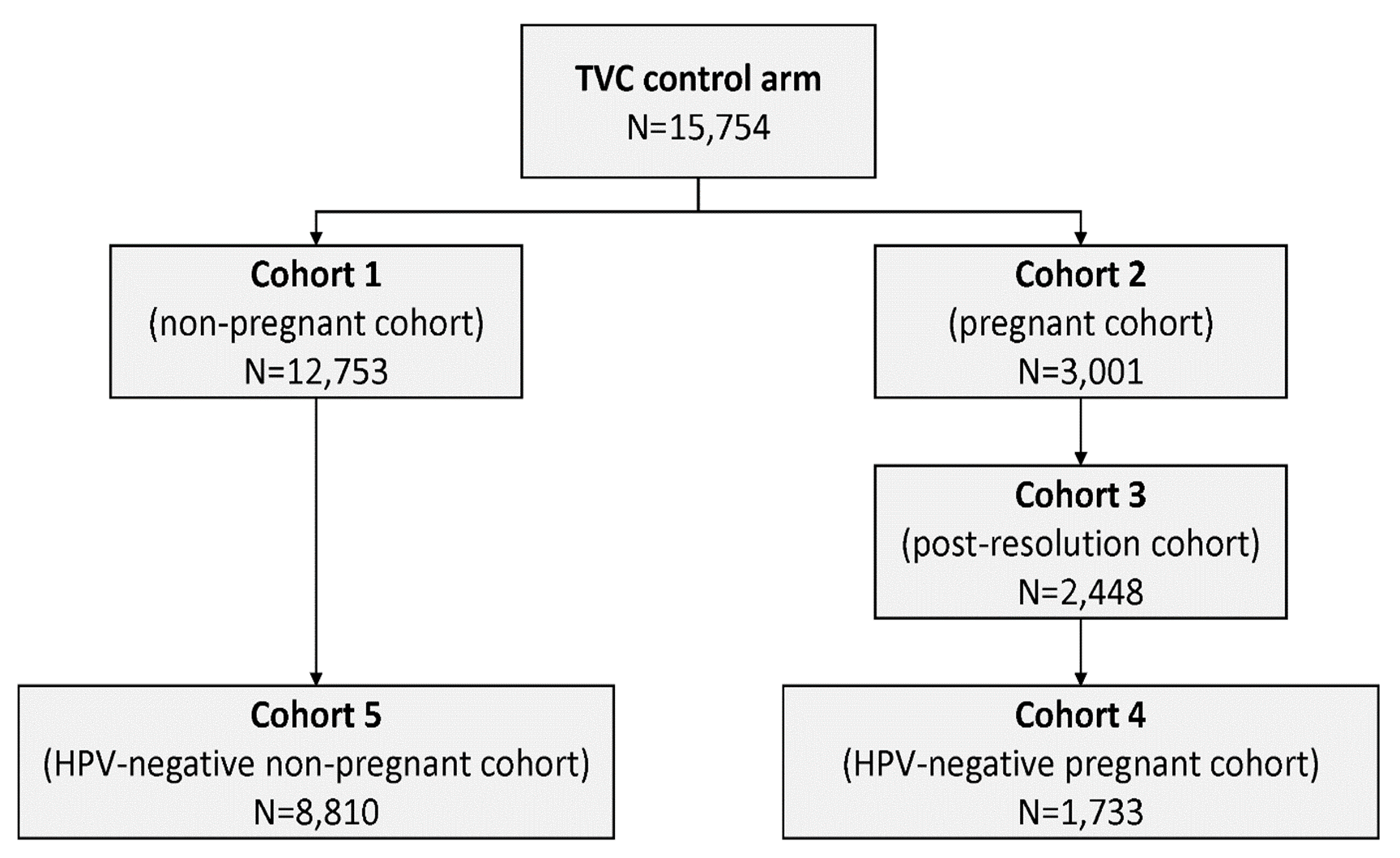
**

**Supplemental Figure 2 Incidence of cervical HPV-positive infection post-resolution of pregnancy, by duration between pregnancy resolution and the first positive post-resolution sample collection (HPV-negative pregnant cohort).** HPV: human papillomavirus; hr-HPV, high-risk HPV.


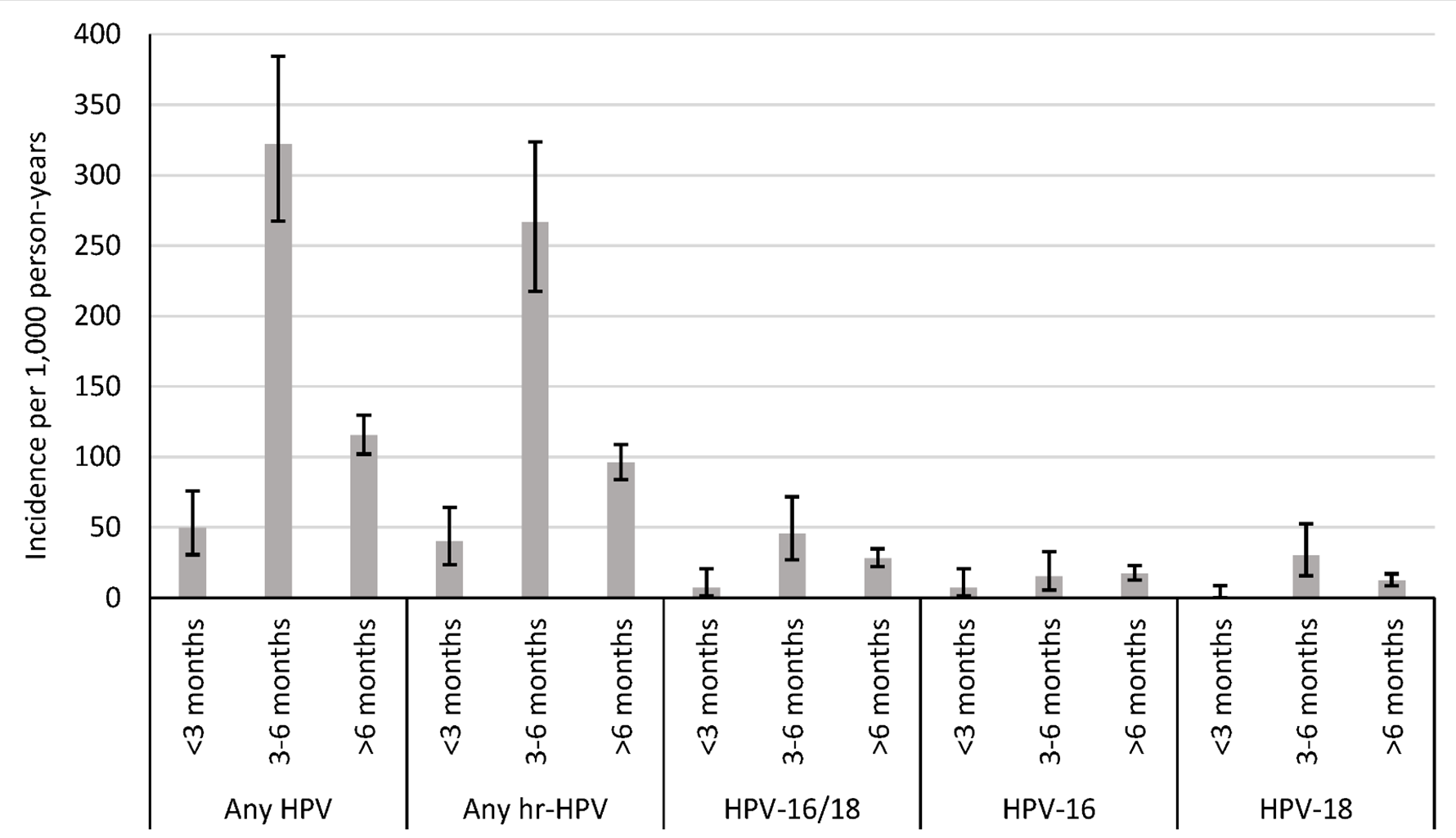


**Supplemental Figure 3 Plain language summary**


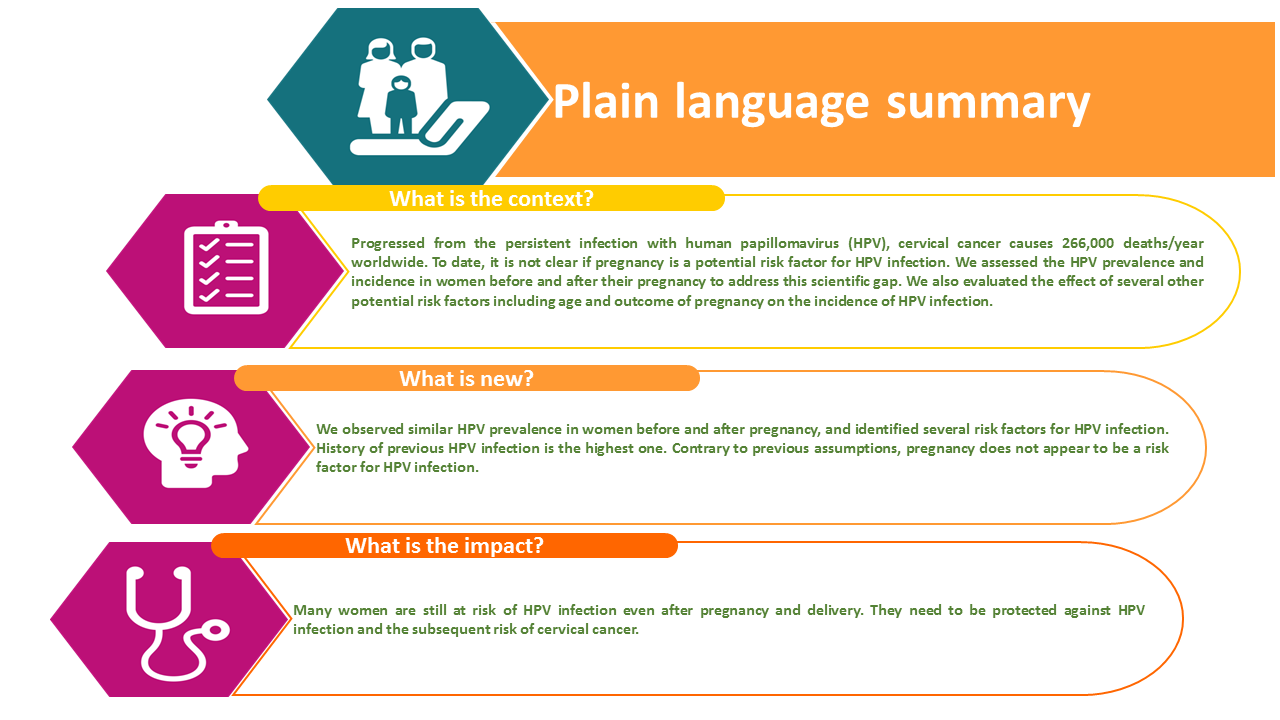

Supplement: ofz486_suppl_Supplementary_Material [file ofz486_suppl_supplementary_material.docx]
